# Supplementary material for: Are carbon emissions trading and green financial instruments synergistic? -Comprehensive quantitative research based on content analysis
Source: PLoS One. 2024 Mar 7;19(3):e0298601. doi: 10.1371/journal.pone.0298601 (PMC10919601; doi:10.1371/journal.pone.0298601)
Supplement: S1 Data — (ZIP) [file pone.0298601.s001.zip › Data/地级市Z_F工作报告.zip!/║┌┴·╜¡/╞δ╞δ╣■╢√╩╨/└┤╫╘╣1⁄2╓┌║┼í╛┬φ┐╦╩2╛▌═°í┐_2012.docx]

各位代表：

　　现在，我代表市人民政府，向大会报告过去五年的工作，对今后五年政府工作目标及2012年工作提出建议，请予审议，并请各位政协委员提出意见。

　　一、过去五年工作的回顾

　　第十四届人民代表大会第一次会议以来的五年，是我市经济社会发展较快的五年。2011年，预计地区生产总值突破千亿元，实现1080亿元，比2006年增长84.7%，年均增长13.1%。完成财政总收入141.3亿元，比2006年增长2.9倍，年均增长31.3%；地方财政收入104.5亿元，比2006年增长3.8倍，年均增长36.9%。全社会固定资产投资完成500亿元，比2006年增长3倍，年均增长31.8%。

　　——这五年，是传统产业不断壮大，新兴产业加快发展，工业结构进一步优化，工业实力和后劲明显增强的五年。2011年规模以上工业完成增加值320亿元、主营业务收入915亿元、利税103.8亿元，分别比2006年增长2.1倍、1.8倍和2.4倍。五年来，实施重点工业项目398项，超亿元项目153项，超10亿元项目11项，完成投资207亿元。一重、北钢、齐轨道交通装备、齐重数控、齐二机床、昊华、飞鹤、博天、博城等老企业的技改、扩能项目顺利实施，一拖东方红大农机、龙华新能源汽车、蒙牛乳业、中粮玉米深加工、中恒药业生物医药产业园等大项目成功引进建设。园区建设“15211”工程进展顺利，在建项目31项，总投资612亿元，完成投资61亿元。齐齐哈尔高新区、交通运输装备制造产业园、蒙西建材工业园等重点园区建设顺利推进，各县园区初具规模。加强与企业总部联系，有力支持了企业发展。

　　争取批次用地指标832公顷，其中后两年争取725公顷，保障了项目建设用地需求。实施非公有制经济（中小企业）培育成长工程，促进了民营企业发展。科技创新成果显著，获得省级以上科学技术奖82项。高新技术企业实现产值280亿元，比2006年增长3倍。我市被命名为国家商标战略实施示范城、国家新型工业化装备制造产业示范基地。节能减排任务全面完成，城市空气质量二级以上天数稳定超过340天。

　　——这五年，是五谷丰登、六畜兴旺，农民顺心、农村祥和，农业农村全面发展的五年。各项惠农政策全面落实，种植业连续四年获得大丰收，农村基础设施不断完善，农民的就医、子女就学、进城务工条件明显改善，农民的心更顺了、收入更多了、生活更好了。2011年粮豆薯总产215亿斤，比2006年增长61.3%，创历史新高；实现农业总产值271.6亿元，比2006年增长40.7%。畜牧业实现产值173亿元，比2006年增长38.7%；奶牛存栏62.2万头，鲜奶总产量145.3万吨，比2006年增长64.9%和77.2%。累计转移农村劳动力107万人，人均劳务收入2994元，比2006年增长3倍。农村居民人均纯收入达到7391元，比2006年增长2.8倍。扶贫开发成效显著，农村贫困人口比2006年下降55%。建设农村饮水安全工程1670处，解决了农村85.7万人饮水不安全问题。改造农村泥草房1416.5万平方米，农村砖瓦化率达到70.6%，比2006年提高18个百分点。种植结构不断优化，玉米、水稻和马铃薯种植面积分别达到1618万亩、339万亩和207万亩，牧草、杂粮和蔬菜种植面积达到210万亩，绿色作物种植面积占耕地总面积的35%。制定了马铃薯产业发展规划，新建千吨以上标准马铃薯贮藏库（窖）78个，贮藏能力增加32万吨，开通了马铃薯营销专列，促进了农民与市场有效对接。兴十四现代农业示范园区达到省级标准，县级农业科技园区发展到37个。投资163亿元推进重点农业产业化项目81项，市级以上龙头企业发展到190户。农民专业合作社由2006年的523个发展到现在的3610个。组建千万元现代农机专业合作社160个，占全省的29.3%，综合机械化程度达到89.2%。土地规模经营面积达到村集体耕地总面积的41.5%。五年来投资27.9亿元，加固堤防51公里，除险加固病险水库27座，实施大型灌区续建配套及节水改造3处，新建灌区渠首1座；新增高效节水灌溉173万亩、水田177万亩；治理水土流失195.5万亩。土地整理完成投资13.6亿元，新增耕地11.9万亩，耕地保护工作连续五年获全省先进。

　　——这五年，是市场体系逐步完善，旅游业加快发展，金融支撑能力明显增强，第三产业不断升级的五年。2011年完成社会消费品零售总额417亿元，进出口总额10.3亿美元，比2006年增长1.3倍和6倍。绿博会、小交会和关东文化旅游节的影响力不断扩大，餐饮、娱乐等传统服务业快速增长。以建设区域性现代物流中心为目标，谋划建设了综合物流产业园区。在扎龙景区建设了生态放飞广场、木栈道、观光水路等项目，在齐扎公路沿线规划了文化旅游节点项目，增强了扎龙景区对旅游业发展的带动力。罗西亚大街、劳动湖、明月岛等重点景区建设扎实推进。与大庆、黑河建立城际旅游联盟，提升了“情系黑土地”旅游线路吸引力。金融业健康发展，居民储蓄存款稳定增长，各类贷款发放全省领先。积极引进浦发银行、辰能风险投资、中国平安投资、中兆广信担保等金融机构，龙江银行县域网点布局全面铺开。成立了26家村镇银行和小额贷款公司。城投公司为大项目建设融投资45亿元，中小企业、畜牧业和马铃薯产业融资平台作用得到有效发挥。

　　——这五年，是城市功能日趋完备，人居环境显著改善，城乡面貌发生深刻变化的五年。五年来，新开发住宅小区64个，建设商品房730万平方米，市区人均住房面积达到25.6平方米，比2006年增加4平方米。从2008年起拆迁城市棚户区518万平方米，占总量的76%；建设回迁房514万平方米，8.3万户居民迁入新居，棚改工作在全国介绍了经验。市区建设廉租房10328套、公租房2300套。改造城市旧小区10个、低温楼57万平方米，启动实施9个城中村改造。以政企共建方式启动了齐齐哈尔新城（中汇城）建设，完成拆迁41万平方米，已有7个项目开工建设。劳动湖南扩、中华路总体风貌改造等重点工程有序推进。公路建设实现历史性突破，4条高速公路、3座跨江大桥以及中断10年的齐富公路建成通车。改造城区主次干道29条，人均道路占有面积达到8平方米。建设农村公路1.7万公里，通乡、通村公路硬化率达到100%和98%。投资20.5亿元开工建设“三供两治”项目35项，供气、供热、供水能力有了新提高。出租汽车行业实行公司化经营初显成效。列入松花江流域水污染防治规划项目如期投入使用。新增城区绿地2413公顷，新建公园游园14个，绿化覆盖率提升到40%，达到了国家园林城绿化标准。植树造林160.3万亩，造林绿化三年会战任务如期完成。乌裕尔河自然保护区晋升为国家级保护区。高标准推进24个省市级试点镇规划建设，实现12个整乡（镇）和127个整村推进，省级示范村达到120个。大力创建“三优两提升”文明城市，集中开展城市重点部位环境综合整治会战，形成了城市管理长效机制，荣获省级卫生城称号。出台了城市规划控制规定，新编和完善了土地利用总体规划、中汇新城、扎龙景区等各类规划567项，为今后城市建设提供了刚性保障。

　　——这五年，是经济社会统筹发展，公共服务能力明显提高，社会事业不断进步的五年。累计完成教育投入127.8亿元，新建和改扩建公办幼儿园61所，建设义务教育标准化学校186所，改造危房校舍100万平方米。扩大了普通高中公费生招生比例，促进了教育公平。中职毕业生就业率达到95%，培养了5.5万名专业技能型人才。齐齐哈尔大学通过国家验收，齐齐哈尔工程学院挂牌成立。深化医药卫生体制改革，有效化解了群众看病难、看病贵问题。131个乡镇卫生院、35个社区卫生服务中心实行基本药物零差率，药品价格平均下降38.8%。投资3.7亿元新建9家县医院、4家中医院，基层医疗保障能力明显提高。市精神卫生中心、第一医院门急诊病房楼、结核病医院等项目建成并投入使用，区域医疗救治服务中心地位进一步提升。在全国率先实施生殖健康促进计划，人口自然增长率控制在4‰以内。我市荣获第六次全国人口普查国家级先进单位。文化事业繁荣发展，创作了以《风刮卜奎》、《神鹤丹丹》等为代表的一批具有地方特色和时代精神的文艺作品。以龙沙公园、嫩江公园内群众合唱团和以兴趣为中心的社区协会组织为代表的群众性文化活动蓬勃发展，极大地丰富了群众的文化生活。和平广场、江桥抗战纪念馆等成为重要的爱国主义教育基地。农村文化惠民五项工程进展较快，国家级历史文化名城建设稳步推进。建设全民健身工程634处，70%的社区配备了全民健身器材，环劳动湖全民健身长廊、沿江大道成为群众健身运动的集中场所。速度滑冰竞赛项目在国内居于领先地位。

　　——这五年，是民生工程扎实推进，城乡居民收入增长较快，人民生活明显改善的五年。城镇居民人均可支配收入达到1.49万元，比2006年增长87.2%。为45376人次发放小额担保贷款12.88亿元，被列为省级创业型城市。累计实现城镇新增就业61.2万人，城镇登记失业率控制在3.9%以内。城镇基本养老、医疗保险参保人数达到41.8万人和171万人，新农保试点和新农合参保率达到91%和99.6%。建立了被征地农民养老保险制度，“五七工”、“家属工”和农林牧渔场职工全部纳入养老保险，启动实施了城镇居民养老保险试点工作，解决了政策性和依法关闭破产国有企业退休人员医保、“老工伤”人员旧伤复发医疗费以及国有企业职工历史工资拖欠问题。职工和居民住院报销比例分别高于全省平均水平14和23.3个百分点，切实减轻了参保人员就医负担。城乡低保应保尽保，五保集中供养率达到51%。2.4万贫困残疾人实现脱贫。“惠民行动”发放就业技能培训、体检、旅游、生活救济等消费券5.27亿元，惠及困难群众102万人次。加快社会养老服务体系建设，被授予全国唯一的“国际老年友好型城市”，龙沙公园成为全国首家“孝亲敬老”主题公园。荣获全国双拥模范城“六连冠”。以保障和改善民生为重点的91件实事基本完成，旧小区低温楼改造、公交线路延长运营时间、中心城区中小学配送营养午餐、校园配备保安等受到了广大群众普遍欢迎。

　　——这五年，是社会管理逐步加强，社会更加安定和谐，人民群众安全感和幸福感不断提升的五年。连续五年被评为全省社会治安综合治理优秀市地，成为全省首批“平安市”，建华区和富裕县被评为全国“平安县（区）”。在全省率先建设报警服务中心，安装监控头12.6万个、智能卡口系统114处，城市技防水平居全省前列。完善“城区大巡防”长效机制，推进警力下沉，破案率明显提高，治安形势持续好转。持续开展打非治违和隐患排查治理专项行动，各类安全生产事故逐步下降。健全市场监管长效机制，开通12315“一线通”受理平台，立案查处各类违法违规案件570件。组织开展了综合治税、收缴陈欠土地使用税和出让金、规范房地产开发企业所得税、清理建筑容积率补缴土地出让金、加强建筑用砂石税费征管等专项治理活动，收缴各种税费3亿多元，其中2010年以来收缴建筑用砂石开采税费7430万元，经济秩序进一步规范。实行社会稳定风险评估制度，强化源头治理，充分发挥“大调解”工作体系作用，妥善办结一批信访积案、难案，信访总量和集体访、越级访数量连年下降。实施全市社区公益用房三年发展规划，近两年投资1.58亿元，新建、改建社区公益用房4.28万平方米。社区服务项目拓展到11个大系列140多个种类，社区事务实现了“一站式服务”。实行物业管理的小区全部建立了业主委员会。提高了社区工作者生活补贴和政治待遇。

　　——这五年，是政府职能加快转变，行政效能不断提高的五年。自觉接受市人大和市政协监督，人大代表议案和政协委员提案办结率均达100%。起草审修地方性法规10部，制定出台政府规章21部。围绕行政审批、工程建设、土地出让、资源管理、城市管理、征地拆迁补偿、财政资金使用、服务项目建设、政府议决事项等方面，制定并实施了20余项制度规定，规范了行政权力运行，优化了行政审批流程。行政监察得到加强，围绕工程招投标、征地拆迁等加大监察力度，严肃查处了一批越权审批、重复审批、虚假招标、乱收费等违法违规案件。大兴调查研究之风，政府系统调研工作取得新成果。政府机构改革稳步推进。积极打造“阳光政务”，加强了政府门户网站建设。开通了行风热线，开设了电视直播互动栏目《关注·沟通》，群众的一些实际问题和困难得到较好解决。开展“大走访”开门评警活动，进一步密切了警民关系。创新开展了领导干部生态审计、建设用地容积率审计和企业税费缴纳审计等专项审计。廉政建设和行政执法责任制深入落实。

　　外事侨务接待、民族宗教、机关事务、关心下一代、地方志、档案、人防、气象、民兵预备役等工作取得了新成绩。

　　各位代表！回顾过去五年的工作，成绩来之不易。这得益于省委、省政府和市委的正确领导，得益于市人大、市政协及各民主党派的监督支持，得益于驻齐中省直单位的鼎力相助，得益于全市各级政府和广大干部群众凝心聚力谋发展，开拓创新解难题，坚定不移促落实。在此，我代表市政府，向全市人民，向所有为我市经济社会发展做出贡献的同志们、朋友们，表示崇高的敬意和衷心的感谢！

　　在肯定成绩的同时，我们也清醒地看到，全市经济社会发展和政府工作中还存在一些困难和问题。新兴产业刚刚起步，产业结构有待进一步优化，牵动发展的战略性大项目还不多。品牌农产品商品量不大，农村城镇化水平不高，农民进入市场的组织化程度不够。现代服务业发展较慢，融资难和运输瓶颈仍是制约经济发展的重要因素。市场发育相对滞后，建设水平较低，监管力度需要进一步加大。就业和社会保障压力较大，加强和创新社会管理任务繁重。个别部门在为经济社会发展创造公平、高效的服务环境上还有一定差距，少数领导干部和公务人员谋发展、抓落实的意识和能力不强。对此，我们一定高度重视，认真加以解决。

　　二、今后五年政府工作目标和2012年工作的建议

　　刚刚闭幕的中国共产党齐齐哈尔市第十二次代表大会，明确了今后五年工作的总体思路和奋斗目标，为做好今后的政府工作指明了方向。政府工作要全面贯彻和体现市委对今后五年工作的总体部署。今后五年的奋斗目标是：

　　——经济健康快速发展。地区生产总值、财政收入比2011年翻一番以上，综合经济实力在全省继续保持前列。

　　——生活水平持续提升。城镇居民人均可支配收入和农村居民人均纯收入实现大幅增长，努力超过全省平均水平。

　　——社会建设更加和谐。社会事业全面加强，民主法制更加健全，社会管理制度不断创新，平安鹤城、法治鹤城、和谐鹤城建设成效明显。

　　——文化建设繁荣发展。社会主义核心价值体系建设深入推进，文化事业全面繁荣，文化产业加快发展，人民群众幸福指数大幅提升。

　　——城乡面貌明显改观。新城开发与老城改造同步推进，城市功能更加完善，人居环境更加优越，特色更加鲜明。新农村建设和小城镇建设迈出坚实步伐，城乡一体化进程进一步加快。

　　——生态建设成效显著。加快建设资源节约型、环境友好型社会，晋升国家级园林城市，可持续发展能力进一步增强。

　　各位代表，2012年是新一届政府工作的开局之年，也是实施“十二五”规划的关键之年。我们要按照市第十二次党代会总体部署，牢牢扭住经济建设不放松，把改善民生作为一切工作的出发点和落脚点，以解放思想为先导，以改革创新为动力，推动全市经济社会又好又快、更好更快发展。今年的主要预期目标建议为：全市地区生产总值增长12%，地方财政收入增长2%，全社会固定资产投资增长30%，规模以上工业增加值增长10%，社会消费品零售总额增长17%，城镇居民人均可支配收入增长12%，农村居民人均纯收入增长13%，城镇登记失业率控制在4.5%以内，万元GDP综合能耗下降3.5%以上。

　　上述建议目标的提出，既考虑了当前不利因素的影响，又考虑了我市发展具备的有利条件。从不利因素看，目前后金融危机影响逐步加深，国内经济增速放缓，物价仍在高位运行，滞胀会在一定时期内存在，经济发展形势不容乐观。我市装备制造、冶金、化工行业趋于严峻，市场萎缩，订单减少，效益下滑；农产品市场竞争日趋激烈，加之部分农产品关税下降，对于市场体系尚不健全、品牌优势尚不明显、产品附加值相对较低的我市来说，将在农产品生产与销售上受到严重影响。从有利条件看，随着国家进一步振兴东北老工业基地战略的实施、积极财政政策和稳健货币政策的实行、对小微企业扶持政策的出台以及省委省政府对战略性新兴产业、基础设施和民生工程投入力度的加大，我市加快发展的宏观政策环境将更加有利；辩证地看，虽然一些企业订单减少，但同时也为企业休养生息、加快技术改造和新产品研发、更好地参与新一轮市场竞争提供了难得机遇；随着国家房地产市场调控力度的加大，一些大型开发企业借助当前建材价格低位运行以及我市商品房刚性需求较大的有利条件进驻我市，这对加快城市开发建设、优化住房结构、扩大住房供应、合理调控房价、推进城市化进程极为有利，必将带动相关产业加快发展；农业连获丰收，以水利为重点的基础设施建设投入不断加大，抗御自然灾害能力增强，为农村经济稳步发展创造了条件；城乡居民收入稳定增长，消费需求持续旺盛，将有力促进第三产业发展；一批产业项目和重大城市基础设施项目开工建设，将有力拉动固定资产投资增长；特别是经过多年来的发展实践，全市上下形成了人心思进、干事创业的良好氛围，发展的信心和动力明显增强。总体看，今年依然是机遇与挑战并存，机遇大于挑战。为实现上述目标，我们要重点做好以下工作：

　　（一）以实施园区建设“15211”工程为重点，全力推进大项目建设，促进工业经济总量扩张、效益提升和发展方式转变。打好产业项目攻坚战。实施重点项目200个以上，确保完成投资200亿元以上。着力抓好一拖大农机、新能源汽车、北满特钢系统优化升级扩能改造、齐重数控重大装备产业化基地、飞鹤鲜奶处理、中粮玉米深加工、泰来及富拉尔基油田开发、龙江铜矿开发等144个产业大项目建设，力争早日达产达效。加快华能煤制天然气、齐化500万吨炼油、风力发电、生物质发电等项目谋划，争取尽快开工建设。搞好太阳能、地热、秸秆等资源综合开发利用，培育壮大新能源产业。加快推进中恒生物制药产业园建设，培育发展新医药和生命科技等相关产业。发挥装备制造研发优势，积极引进新技术新成果，发展新材料产业。争取国家重大科技专项、省重大产业结构调整专项10项以上。提高园区建设水平。统筹搞好园区产业规划和功能区划，错位发展，突出特色，提高对项目的承载能力。齐齐哈尔国家高新区及交通运输装备产业园实行“一区五园”布局和管理，着力发展高端服务、科技研发、总部经济和交通运输产业。富拉尔基工业园区以打造国家高端装备制造业研发生产基地和装备制造配套产业基地为重点，着力引进大型和产业链长的项目。北苑工业园区立足于打造全国大马力拖拉机生产基地和现代农机制造交易中心，加快完善基础设施和引进配套企业，启动并推进兵器工业园建设，争创省级开发区。铁锋产业园区以打造黑龙江省西部地区最大的现代物流中心为目标，重点推进物流项目及专用汽车生产基地建设。昂昂溪工业园区以石油化工为支柱产业，打造上下游联系紧密的石化产业链。坚持项目建设优先，政策、资金跟着项目走，加快建设富裕轻工食品工业园、依安陶瓷产业园、龙江齐西经济开发区、讷河希望工业园、甘南兴十四现代农业示范园、克东蒲峪路工业园等一批县域特色园区。坚持园区基础设施与项目建设同步推进，实行滚动式开发，为项目进驻创造条件。严把项目质量关，优先选择投资额度大、科技含量高、税收贡献多、拉动能力强、环境污染小的项目入园，避免低水平重复建设。加大服务支持企业力度。发挥政策的引导作用，支持企业核心业务与非核心业务分离，促进不同所有制企业在同一产业链条上协作配套，推动关联度高的企业集聚发展。围绕大企业和大项目搞好配套产业规划和定向招商，培育壮大产业集群。继续加强与企业总部联系，力争在资金注入、项目建设、市场开拓上获得更大支持。坚持抓大带小，积极辟建中小企业园区，扶持科技型企业发展。创建中小企业服务平台，加强融资服务，支持企业上市。优化审批服务流程，对重点项目给予保姆式跟踪服务，积极帮助企业解决各种困难。

　　（二）大力发展现代农业，扎实推进新农村建设，加快城乡一体化进程。以建设现代化大农业为统领，以建设国家重要的农产品生产、加工和营销基地为目标，扩大中国绿色食品之都的影响力。突出抓好农业产业化经营。由抓“产加销”向抓“销加产”转变，积极培育优质绿色农产品知名品牌，加快发展专业营销主体，瞄准大城市、大市场、大超市，减少中间销售环节，扩大直销比重。坚持围绕农业上工业，引进发展一批成长性好、带动力强的农产品加工企业，加快农产品生产大市向加工大市跨越。巩固提高粮食综合产能，大力发展高产、优质、高效作物，新建国家级绿色食品原料基地50万亩，玉米、水稻种植面积分别达到1700万亩和360万亩，确保粮食产能稳定在200亿斤以上。坚持突出抓种薯、着力搞贮藏、加工促转化、外销拓市场，大力发展马铃薯产业，种植面积达到215万亩，其中种薯面积30万亩，叫响种薯品牌，做大种薯经济。抓好“菜篮子”工程，加快梅里斯等蔬菜生产基地建设，力争达到60万亩。大力发展杂粮产业，种植面积达到135万亩，打造全国杂粮产业基地。努力提升农业科技化水平。加快普及推广农作物优质专用品种，优良品种覆盖率达到98%以上。搞好主要农作物的高产攻关、栽培模式研究和品种选定，重点推广10项农业新技术，主要农作物标准化率达到90%以上。加快兴十四国家级现代农业示范园区建设，建立完善县、乡、村三级科技园区。积极向上争取粮食和蔬菜生产的科技推广项目，全面提升科技支撑能力。着力提高农民组织化程度。抓住畜牧业发展不放松，以提高畜牧业组织化程度和品牌效益为重点，全面推进主要畜禽品种规模化、标准化、产业化发展。突出抓好奶牛业发展，鼓励和扶持规模养殖，奶牛存栏达到68万头，其中规模养殖39万头；鲜奶产量达到160万吨。提高畜禽养殖科技含量，促进畜牧业发展提档升级。抓好畜禽疫病防控工作，严防重大疫情发生。加强农民专业合作社建设和管理，扩大经营合作领域和规模，新发展各类专业合作社390个。抓好千万元现代农机合作社项目完善提高和争取配套建设，逐步实现粮食作物全程机械化。培育土地使用权转让市场和社会化服务体系，促进土地集中使用和生产要素向土地集中投入，土地规模经营达到1320万亩。积极推进农田水利化建设。抢抓国家和省加大农田水利建设投入的有利机遇，完成泰来抗旱灌溉引水工程建设和35座病险水库改造，加快三大灌区续建配套与节水改造及尼尔基水库下游配套工程建设，积极争取龙江花园水库枢纽、尼尔基直供灌区等10个工程立项开工。抓好西部地区节水灌溉、东部地区水资源开发，全面推广节水工程新技术，新增水田20万亩、节水喷灌50万亩。治理水土流失45万亩。加快实施城乡一体化发展。积极推进省市级试点镇规划建设和城中村改造，完善配套设施和服务功能，统筹解决好进城农民的户口、就业、收入、保障等问题，增强产业集聚和吸纳人口能力，争取城市化率提高2个百分点。鼓励支持各县（市）区抓好整乡（镇）和整村推进工作，编制重点村建设和发展规划，逐步撤并自然屯，加强小城镇和中心村建设，大力整治村容村貌。建设农村公路3860公里，改造农村泥草房3.86万户，植树造林25.5万亩。积极发展农村清洁能源。累计转移农村劳动力110万人，劳务收入增长9%以上。

　　（三）加快发展现代服务业，壮大第三产业规模，培育新的经济增长点。做大做强旅游产业。深入挖掘旅游资源，丰富旅游文化内涵，在城市建设中突出旅游功能，全面提升旅游业的影响力和带动力。重点建设好扎龙景区及齐扎公路文化旅游节点，开工建设扎龙景区入口综合区、湿地博物馆项目，积极推进扎龙旅游名镇建设，重点抓好温泉酒店、老年大学、温泉度假别墅区等项目，加强齐扎公路沿线环境整治。搞好昂昂溪罗西亚大街、碾子山自然山水、梅里斯民族风情等旅游资源开发，着力打造城市近郊和县区特色旅游。加强区域旅游联盟合作，对旅游资源和产品进行综合推介，支持旅行社发展壮大，增加至北京、大连、深圳等地航班，带动旅游业发展。加快发展商贸流通业。围绕社会发展和人民群众对文化、旅游、运动、休闲、娱乐、购物的需求，以中汇城建设为载体，积极发展新型商业业态，不断完善老城区商业服务功能，统筹发展全市商贸流通业。铁锋现代物流园区年内启动建设70万平方米，汽贸城新引进4S店9家，争取5年内园区全部建设完成。加快各区县物流项目建设，构建一个综合园区、多个专业中心的物流体系。积极引进和发展大型物流企业集团，搭建公共信息平台，推动物流业集群化发展。加快解放门市场等传统市场改造和提档升级，规范壮大专业市场，增强市场辐射能力。支持金融业发展和融资平台建设。积极引进外埠金融机构，搞好金融业发展布局。推进企业上市融资，促进产业资本和金融资本融合。推进诚信体系建设，营造诚信的金融环境。做实做强城市投融资平台，规范城市投融资管理。加快信息、中介、服务外包等生产性服务业发展，增强第三产业支持区域经济发展的能力。

　　（四）全力推进新城建设，统筹搞好老城改造，彰显开阔疏朗、自然亲和、整洁优美的城市特色。我市城市化水平低是现实的差距，也是发展的潜力。我们要以加快城市化进程为主线，坚持新城开发与老城改造相结合，小城镇建设与城中村改造同步推进，把生态、交通和就业优先贯穿城市规划建设始终，突出城市特色，彰显城市品位，提升集聚能力，打造宜居宜业型区域中心，争取用5年左右时间城市化率达到全国平均水平。搞好城市住房建设。坚持总量控制、有序开发、统筹兼顾、优化结构的原则，促进房地产市场持续健康发展。推进房地产业全方位对外开放，引进有实力、有信誉的大型开发企业入驻，支持本地大型建筑企业发展，快速提升城市建设品位。统筹搞好商品房开发、城中村改造和保障性住房建设，满足各层面住房需求。有序供应建设用地，把商品房价格控制在合理区间，让城乡居民买得起房、住得起房，吸引农村人口向城市聚集。今年市区开发建设商品房200万平方米，新开发中汇城地段、溪水片区等一批住宅小区。加快保障性住房建设，改造城市棚户区85万平方米、建设回迁楼68万平方米，建设廉租房300套、1.35万平方米，建设公租房400套、2.2万平方米，力争1万户居民迁入新居。改造旧小区10个，改造低温楼50万平方米。提高采暖标准并加强供热质量监管，确保达到19摄氏度以上常态温度。加快城市重点工程建设。主要围绕“一城三线”展开。“一城”，就是以建设“高新产业园区、现代商贸都会、休闲度假乐园、生态宜居新城”为目标，全力推进新城建设。加快建设公安指挥中心、中级法院审判中心、一重总部和研发中心、哈齐铁路客运新站、公路客运站等15个公建项目，完成主要道路及配套设施建设；积极推进高端住宅及配套开发、星级酒店及酒店式公寓、新型时尚商业步行街、一站式购物公园、综合商业展示交易中心、时尚家居生活馆等项目，打造新一代城市综合体。力争用5年时间建成可以容纳30万人口的城市副中心。“三线”，就是建设劳动湖南扩这条“通航滨水线”，完成河道、船闸、桥梁、人行道、亮化和绿化工程建设，搞好休闲商务区、特色美食街、沿江风光带建设，打造集生态、居住、旅游于一体的滨水景观区；建设齐扎公路文化旅游节点及扎龙景区这条“品牌旅游线”，着力提高城市的旅游知名度和竞争力；建设中华路总体风貌改造这条“迎宾门户线”，突出历史风貌和生态特征，加快建成迎宾窗口。抓好城市道路、“三供两治”、绿化等市政工程建设。加快主次干道和巷道改造，推进合意大街、军校街等7条重点街路建设。围绕新城建设启动城市南环路建设项目。加强公路养护管理，保护公路建设三年决战成果。坚持公交优先发展战略，科学规划设置中心城区公交线路和公交站点，继续增加公交延时线路。进一步深化出租汽车行业管理改革，全面提升出租车管理服务水平。加快城区供水工程建设，改造3座二次供水泵站，新建二次供水管网8000米。抓好城南热电联产一期工程，提高城区供热能力。启动昂昂溪区天然气利用工程，铺设燃气管线8公里。推进城区污水管网扩建二期工程，改造铺设重点路段雨水管网5000米，新建雨水泵站1座。完成城区垃圾处理厂建设。加大新城绿化力度，打造一批绿化样板。对老城区14条主要街路、5个公园游园、6处广场绿地进行绿化升级。提升城市管理效能。实施“大城管”综合执法，完善“两级政府、三级管理、四级网络”管理体系。推进城市市场化、精细化、群众化和人性化管理，探索数字化城市管理的有效途径。今年春季开始集中开展城市管理专项会战，大力整治交通秩序、环境卫生、私搭乱建以及牌匾广告、路边工厂、路边烧烤，综合整治渣土等运输车辆造脏、超载、超速、乱卸等行为，建立长效治理机制，巩固“三优两提升”文明城创建成果。

　　（五）加快社会事业建设，推动文化大发展大繁荣，促进公共服务均衡协调发展。加快发展文化事业和文化产业。坚持走出去与请进来相结合，以大众文化为根基，以精品文化为灵魂，从培养青少年的文化素质抓起，积极构建核心价值体系，突出地方文化、民族文化和历史文化特色，推动文化与旅游相结合，促进文化发展由基础向高端、由高端向产业迈进，不断提高城市的文化吸引力和群众的文化消费水平，让广大人民群众感受文化带来的快乐。深化文化体制改革，提高公共文化服务能力和水平。大力发展公益文化事业，加快工人文化宫和马戏团改造、图书馆新馆、群众艺术馆新馆、青少年活动中心、复建黑龙江将军府等重点文化项目建设，将市图书馆新馆建成集城市规划展示馆、电子图书馆、豪华影剧院和售书、阅览于一体的新型图书馆。全市图书馆、群众艺术馆（文化馆）、博物馆、文化站等156处公共文化设施全部免费开放。新建乡镇文化站14个、农家书屋335个，农村文化五项惠民工程实现全覆盖。大力开展群众性文化活动，积极发展以兴趣为中心的社区协会组织。发挥群众性合唱团作用，繁荣活跃广场文化和休闲文化。根据人口分布情况，搞好城乡影剧院规划建设。以宣传塑造城市形象为主线，抓好文艺精品生产，支持《中国的中》、《血战嫩江桥》等影视文艺作品完成创作。扎实推进国家级历史文化名城建设，做好文物和非物质文化遗产保护工作。加大鹤文化系列产品开发力度，高水平规划建设中国鹤文化产业园，打造一批鹤文化标志性建筑，积极筹办以鹤文化为主题的婚纱摄影、婚礼、寿宴和会展。依托马戏团、克东满绣、嫩江流域玛瑙石资源、梅里斯达族绣刺绣基地和富裕漫画基地，加快发展特色创意文化产业。提升文化创意产业园发展水平，引导人才和项目向园区集聚。抓好文化队伍建设，加大培养引进人才力度。发挥高校作用，鼓励支持高校参与地方文化建设。加强文化市场综合执法，保障文化市场健康发展。努力办好人民满意教育。认真实施全市教育发展规划，加大教育投入，建设义务教育标准化学校47所、“校安工程”30.8万平方米，促进义务教育均衡发展。新建和改扩建公办幼儿园30所，着力发展农村学前教育，加快普及高中阶段教育。推进职教综合实训基地建设，提高毕业生就业能力。鼓励支持高等院校内涵发展，加强校地共建，增强高校服务地方经济发展能力，积极推进师范专科学校升本工作。加快发展民办教育，支持远程教育和继续教育。建立高端优质培训基地，加大教师培训力度，积极推进素质教育，切实提高教育质量，进一步改善师德师风。加强校车、校园周边环境和交通秩序及小饭桌管理，在有条件的学校实行多口出入，确保学生和学校安全。大力发展医疗卫生事业。以提升区域医疗救治服务中心辐射能力为目标，整合市第一医院、中医院等重点医院的医疗卫生资源，加快第一医院南苑一期工程、中医院门急诊病房楼、结核病医院门诊病房楼等项目建设，全面提升医疗和护理水平。建立基层医疗卫生机构运行新机制，提高基层卫生服务效率和质量，村卫生所逐步实现四室一房规范化设置且不低于60平方米。全面实施国家基本药物制度，实行零差率销售。抓好重大传染病防控，提高突发公共卫生事件应急处置能力。为200名贫困白内障患者实施免费复明手术，为500名贫困精神病患者发放免费药品。深入开展全民健身活动，积极开展国民体质监测工作。促进竞技体育发展，力争在国内国际大赛上取得佳绩。统筹做好人口和计划生育工作，对新婚夫妇从登记到子女3周岁全程主动提供科学育儿指导咨询，提高出生人口素质。

　　（六）加强和创新社会管理，突出抓好民生实事，增进广大群众的福祉。针对社会管理中的新情况、新问题和人民群众的新要求、新期盼，我们要切实转变管理的方式方法，整合优化、科学配置社会管理资源，不断提高信息化管理水平；切实办好关系群众切身利益的就业再就业、社会保障、扶贫解困、居住条件改善、食品药品安全、社区建设等11个方面29件实事，把人民群众的安危冷暖时刻放在心上。实施就业优先发展战略。推进创业型城市建设，将促进就业专项资金纳入各级政府财政预算，完善和落实财税、信贷、社保补贴等优惠政策，为5000人发放小额担保贷款2亿元，实现城镇新增就业9万人。实施就业援助，深入抓好高校毕业生、农村转移劳动力和城镇就业困难人员就业工作，帮助3万名就业困难人员实现就业。健全城乡统一的人力资源市场，实现劳动力资源有效配置和合理流动。建立以就业为导向的培训机制，增强就业竞争能力。全面提高社会保障水平。健全覆盖城乡的社会保障体系，完善失地农民养老保障制度，将被征地农民养老保险待遇纳入省级统筹，完成“城中村”改造中被征地农民参保缴费工作。新农保参保率达到80%以上。实施医保城乡一体化管理，将新入校学生全部纳入医保，实现城镇居民基本医疗保险全覆盖。城镇居民基本医疗保险年最高支付限额达到全市居民人均可支配收入的6倍，且不低于5万元。将29万名关闭破产国有企业、集体企业和其他困难企业退休人员以及灵活就业参保人员纳入十二种特殊慢性病保障范围。提高新农合筹资标准和报销比例，新农合参合率稳定在98%以上。抓好集体企业改制工作，落实好国家关于厂办集体企业和其他类型集体企业改制政策，妥善解决职工的劳动关系和社会保险接续及再就业问题。进一步规范城乡低保管理，建立与经济发展水平相适应的增长机制和分类施保制度，确保动态管理下的应保尽保。完善和落实民营养老机构扶持政策，新建百张以上床位民营养老机构5家，提高老年友好型城市建设水平。加快发展社会福利和慈善事业，推进翔鹤养老城、流浪未成年儿童保护中心、社会福利事业从业人员职业技能培训基地等项目建设。推进开发式扶贫，使5.2万人实现脱贫，到“十二五”期末贫困人口下降52%。解决200个村屯、10.5万人饮水不安全问题。加大对贫困革命老区扶持的力度。稳步推进乡（镇）行政区划调整。积极争创全国双拥模范城“七连冠”。全力维护社会和谐稳定。健全社会治安防控体系，继续加大城市技防系统建设投入，加强流动人口、特殊人群和虚拟社会管理，严厉打击各类违法犯罪行为。实行和谐拆迁、人性化拆迁和依法依规拆迁，坚决惩治抢建抢栽、弄虚作假等骗取补偿行为。全面落实安全生产责任制，推进安全生产标准化建设，突出道路交通、建筑施工、危险化学品、消防安全等领域的专项整治，坚决杜绝重特大事故发生。加强应急救援指挥平台建设，建立重大危险源监控和预警系统，提高应急救援处置水平。加强市场监管，维护市场经济秩序，保持物价水平基本稳定。实施食品安全工程，实行注册食品企业和行政执法网上公示制度，选定100家小工厂、小作坊、小饭店对其食品加工过程进行视频监控并上传互联网，设立举报专项奖励资金和110举报电话及网上举报平台。开展医疗和医药市场专项治理，严厉打击虚假宣传、药价虚高、无照经营和非法行医等违法违规行为。严格落实信访工作责任制，健全“大调解”工作体系，推进联合接访大厅建设，做好试点县区的群众工作部组建工作。办好市长热线、行风热线等沟通载体，重点抓好政府门户网站行政审批许可平台和执法平台建设，扩大网上办事服务项目，推进执法情况公开，畅通群众利益诉求表达渠道。进一步加强社区建设。全面完成社区公益用房三年发展规划目标，市区社区公益用房使用面积平均达到300平方米以上，达标率达到100%。深入开展“百项服务进社区”和农村社区“三新”主题实践活动，提高社区服务水平。深化社区管理体制改革，增强社区居委会和业主委员会的服务功能。把今年确定为“物业管理年”，充分发挥业主委员会作用，提高小区物业管理水平，将不负责、不合格的物业企业坚决清出市场。切实抓好村务公开工作。农村社区建设启动率达到60%，建设样板社区10个。

　　各位代表，做好新形势下的政府工作，必须切实加强政府自身建设，努力建设人民满意的政府。从市政府班子做起，带头遵守《廉政准则》，严格按照法律、制度、规则和程序办事，自觉接受市人大法律监督、工作监督和市政协民主监督，始终对群众充满感情，对工作勤勉尽责，对事业毫不懈怠。创新体制机制，抓好流程再造，深化行政审批制度改革，进一步提升政府效能。优化财政支出结构，加强政府投资监管，提高投资效益，把有限的资金用在刀刃上。始终保持干事创业的激情和奋发有为的状态，聚精会神谋发展，全力以赴抓落实，确保政府各项工作落到实处。创新绩效考评方式，深入开展治庸问责，强化执法监察、廉政监察和效能监察，大力整治行政不作为乱作为、工作不配合不落实现象，严肃查处庸懒散漫、违法乱纪、影响政府执行力和公信力的公务人员，树立为民、务实、清廉的政府形象。

　　各位代表！齐齐哈尔正处在加快发展的关键时期和攻坚时期。过去的成绩令人鼓舞，美好的蓝图催人奋进。让我们在市委的正确领导下，万众一心，埋头苦干，为建设富裕文明和谐的新鹤城而努力奋斗！
